# Dietary Energy Levels Affect Carbohydrate Metabolism-Related Bacteria and Improve Meat Quality in the *Longissimus Thoracis* Muscle of Yak (*Bos grunniens*)

**DOI:** 10.3389/fvets.2021.718036

**Published:** 2021-09-22

**Authors:** Mei Du, Chao Yang, Zeyi Liang, Jianbo Zhang, Yayuan Yang, Anum Ali Ahmad, Ping Yan, Xuezhi Ding

**Affiliations:** ^1^Key Laboratory of Yak Breeding Engineering, Lanzhou Institute of Husbandry and Pharmaceutical Sciences, Chinese Academy of Agricultural Sciences, Lanzhou, China; ^2^Key Laboratory of Veterinary Pharmaceutical Development, Ministry of Agricultural and Rural Affairs, Lanzhou Institute of Husbandry and Pharmaceutical Sciences, Chinese Academy of Agricultural Sciences, Lanzhou, China; ^3^State Key Laboratory of Grassland Agro-Ecosystems, School of Life Sciences, Lanzhou University, Lanzhou, China

**Keywords:** yak, dietary energy level, meat quality, *longissimus thoracis*, carbohydrate metabolism related bacteria

## Abstract

The effects of different dietary energy levels on the ruminal bacterial population, selected meat quality indices, and their relationship in yak *longissimus thoracis* (LT) muscle were assessed in this study. A total of 15 castrated yaks were randomly assigned to three groups with low- (NEg: 5.5 MJ/Kg, LE), medium- (NEg: 6.2 MJ/Kg, ME), and high- (NEg: 6.9 MJ/Kg, HE) dietary energy levels and occurred in the cold season (March to May). All yaks from each treatment group were humanely slaughtered and sampled on the day of completion of their feeding treatment. The results showed that the water content and crude fat levels of the LT muscle were markedly elevated in the HE group (*P* < 0.05), while the shear force was drastically reduced (*P* = 0.001). Methionine, aspartic acid, and glycine levels in the LT muscle were higher in the LE group compared with the ME and HE groups (*P* < 0.05). The glutamic acid level in the ME group was greater in comparison to the LE and HE groups (*P* < 0.05), while the histidine level in the ME group was higher than that in the HE group (*P* < 0.05). Additionally, the HE diet significantly elevated (*P* < 0.05) the abundance of carbohydrate metabolism-associated bacteria including *Prevotella_1, Lachnospiraceae_NK4A136_group, U29_B03, Ruminiclostridium_6, and Ruminococcaceae_UCG_013* in the rumen. The results of the Spearman's rank correlation analysis showed that the abundance of *uncultured_bacterium_f_vadinBE97* and *uncultured_bacterium_f_Lachnospiraceae* showed a significant influence on the indicator of IMF and SF. In conclusion, a high dietary energy level improved the meat quality in the LT muscle of yak mainly by increasing the relative abundance of ruminal amylolytic bacteria to provide substrates for fatty acid synthesis.

## Introduction

Yak (*Bos grunniens*), is known as “the treasure of the plateau” and is mainly distributed in the area of Qinghai Tibetan Plateau at an altitude of above 3,000 m (1). It provides more than 90% of the milk and about 50% of the meat consumed in this region (2). Yak meat is a semi-wild natural green food and known as the crown of beef. It is rich in protein, amino acids, carotene, calcium, phosphorus, and other trace elements. However, the yak cannot obtain enough feed supply due to the long-term extensive grazing, breeding mode, and lack of forage biomass during the cold season. This malnutrition state results in a long growth cycle and poor meat quality such as low level of tenderness, intramuscular fat content, and taste (3). With the global rise in the importance of green food and growing consumer demand, the production of high-quality yak meat is of extensive concern domestically and internationally. The use of supplementary feeding could be an effective way to reduce the gap between the production of high-quality yak meat and increased market demand (4). Liu et al. (5) reported that fattening yaks with a total mixed diet in the cold season can improve the yield and quality of the yak meat. Zhang et al. (4) reported that supplementing a diet with high-protein in early-weaned yaks could increase intramuscular fat accumulation. Similarly, Kang et al. (6) demonstrated that the growth performance, meat production, and meat quality of yak could be improved by increasing the dietary energy concentration. These results suggested that the dietary energy concentration has a positive impact on the growth performance, carcass characteristics, meat production, and meat quality of livestock.

Rumen microbiota plays a pivotal role in feed digestion and acts synergistically to degrade plant structural and non-structural carbohydrates into volatile fatty acids (VFAs) and microbial proteins (MCP) (7). The end products of rumen fermentation (VFA) are used as the substrate for fueling other animal tissues (including liver, fat, and muscle) and MCP are required by the host to produce meat and milk (8). The change of rumen bacterial community structure was reported to be closely related to the composition of the diet (9). Dietary components affect rumen fermentation and the structure of the rumen microbial population (9). It has been reported that high-energy diets increased rumen amylolytic and propionate-producing bacteria populations such as *Prevotella, Ruminobacter amylophilus, Succinimonas amylolytica*, and *Bifidobacterium* (10). Feeding high-starch and high-grain diets decreases some fibrolytic bacteria, i.e., *Ruminococcus flavefasciens, Fibrobacter succinogenes*, and *Butyrivibrio fibrisolvens* (11, 12). Lin et al. (13) demonstrated that sheep had a capacity to remodel the structure of the microbiota to adapt to a high-grain diet for a long time; *Ruminococcus, Prevotella*, and *Bifidobacterium* were tolerant to the diet with stable proportions in each treatment in a sheep model. Thus, diet is a main factor affecting rumen microbial diversity, and its nutritional levels or nutrients composition have a significant effect on rumen microbial communities, which may be due to the bacterial preference for feed ingredients, specific metabolites, and rumen environment (14). In addition, improving dietary energy levels is an effective approach to facilitate the growth performance in yaks and the utilization of dietary energy sources depends on the digestion of feed nutrients by rumen microorganisms (15). However, the details of how dietary energy level regulates ruminal microbiota remain unclear.

Intramuscular fat (IMF) and its fatty acid composition play pivotal parts in determining the meat grade for human consumption (16). It has been widely recognized that the meat grade and flavor are intimately linked to the degree of IMF and fatty acid profiles (17). Several studies highlighted that the IMF content in animal meat could be enhanced by providing a high-energy diet (18). The fat content and fatty acids in ruminants are mainly affected by the dietary nutrition and bacterial metabolism in the rumen (19). However, little information is available about the mechanism by which modulatory effects of rumen microbiota contribute to improving the muscle fatty acid profile, amino acids composition, and other quality parameters of yak fed with different levels of dietary energy. Thus, the aim of this study was to investigate the effects of dietary energy levels on the meat quality, rumen bacteria populations, and the relationship between rumen bacteria and meat quality parameters. Moreover, rumen bacteria contributing in the improvement of meat quality on the yak *longissimus thoracis* (LT) muscle would be identified.

## Materials and Methods

### Experimental Procedure and Sample Collection

This experiment was performed at Hongtu Yak Breeding Cooperatives of Tibetan Autonomous Prefecture in Gansu Province, China. A total of 15 adult castrated yaks (initial BW, 276.1 ± 3.5 kg) originating from the local herders were randomly allotted to different energy level treatments, i.e., low energy level (LE: 5.5 MJ/kg), medium energy level (ME: 6.2 MJ/kg), and high energy level (HE: 6.9 MJ/kg). The basic diet composed mainly of 40% oats silage, 40% micro-storage of corn straw, and 20% highland barley hay. Energy levels of three diets met or exceeded the estimated requirements for a 275 kg finishing beef cattle with an average daily gain of 1 kg in the Feeding Standard of Beef Cattle (NY/T 815-2004). The details of the ingredient and nutritional composition for energy diets have been presented in our previous study (20) and briefly summarized in Supplementary Table S1.

All the yaks underwent an acclimatization period of 15 days before study commencement, whereby the designated dietary regimens were implemented accordingly for 60 days. Throughout the study, the animals were individually fed twice daily *ad libitum* and had free access to water provisions. After 60 days, the animals were fasted for 12 h and consequently, humanely slaughtered by electrical stunning. The LT (12th−13th rib) samples were rapidly removed from the carcass regions in quadruplicate, placed within a sterile vacuum packaging, and ultimately stored at 4°C. In addition, LT samples that are required to determine the amino acid/fatty acids (FA) content were stored at −80°C.

### Analytic Methods for Chemical Composition and Amino Acids

The chemical compositions for the LT samples were determined according to Chinese recommended standardized protocols. Water content was determined in line with GB/T 5009.3-2010 through the direct-drying methodology. The crude protein content (Kjeldahl N × 6.25) was determined in line with GB/T 5009.5-2010 through the Kjeltec Auto Analyzer®. The IMF content was determined in line with GB 5009.6-2003 using a Soxhlet Extractor. The crude ash content was determined in line with GB/T 9695.18-2008. The amino acid concentrations were determined based on the previously reported method (GB/T5009.124-2003) (21) using a liquid chromatography (LC, u3000, Thermo Fisher™).

### Meat Quality

The pH value of the LT was determined at 1 h and 24-h post-mortem using a portable pH-meter (PHBJ-260, purchased from INESA Scientific Instrument Co., Ltd.). The pH meter was fitted with a spear tip pH electrode and an automatic temperature compensation probe, and it was calibrated with pH 4.01 and 7.00 buffers in advance. A portable colorimeter (Minolta CR400, Konica Minolta, Japan) equipped with an 8-mm aperture, 10° viewing angle, and D65 illuminant was used to determine the meat color. For each meat sample, five different positions were selected to determine the brightness value (L^*^), redness value (a^*^), and yellowness value (b^*^). Chroma value (H^*^) and color saturation value (C^*^) were calculated based on the L^*^, a^*^, and b^*^ values.

Cooking loss and shear force (SF) were determined according to a procedure adapted from Honikel (22) and Oillic (23). For cooking loss determination, five thawed LT samples that underwent external fat trimming and light blotting for moisture removal were weighed and this readout was recorded as the initial weight. Consequently, the LT samples were heated using a water bath at 80°C up to an internal temperature of 70°C, which was monitored using an internal thermocouple (Eirelec Ltd.™, Ireland). All LT samples were cooled to room temperature, residual moisture was removed using a tissue paper, and weights were measured and recorded as the final weight.

Preparation loss was represented by the final weight/initial weight (%). The prepared LT samples were sliced into five cubes (6 cm ^*^ 3 cm ^*^ 3 cm) and the SF of each cube was determined using a Warner-Bratzler shear apparatus. For water loss analysis (pressing loss), a 10 g LT sample from each animal—wrapped with 12 layers of filter paper—was pressed by a force of 10 kg/cm^2^ for 5 min. Residual moisture was lightly removed and the sample weight was quickly recorded as the final weight. Data were presented as a percentage of the final weight/10.

### Rumen 16s rDNA Sequence Data Analysis

The raw data of the 16s rDNA sequence for rumen bacteria of yaks in the LE, ME, and HE groups were obtained from our previous study (24), which have been deposited in the European Nucleotide Archive (ENA) at EMBL-EBI under the accession number PRJEB34298. The previous analysis was conducted 4 years ago, and the representative sequences of operational taxonomic units (OTUs) were annotated by using the GreenGene database (25) (uploaded in 2013) which led to 30% of the high-quality reads on the genus level not being annotated. Thus, in this study, we re-analyzed the sequence data using an updated database and the details for the analytic methods are as follows. Raw paired-end reads were merged using the FLASH (version 1.2.11) software to generate the contigs and then assigned to each sample according to the unique barcodes (26). The contigs underwent quality control through trimming and filtering by Trimmomatic (version 0.33) with a criterion, i.e., sequences with an average quality <20 over a 50-bp sliding window were rejected (27), then chimeras were identified and removed by the UCHIME (version 8.1) software (28) to obtain high-quality sequences. The generated high-quality sequences were clustered into OTUs by USEARCH (version 10.0) at 97% similarity levels, and the OTUs were filtered when an abundance of <0.005% (29) was observed. We selected the sequences with the maximum abundance in each OTU as the representative sequences using the QIIME (version 1.9.0) software, and the representative sequences of the OTUs were compared with the SILVA database (version 132) using the RDP classifier with a 0.80 confidence threshold (30). After that, Chao1, Shannon and Simpson indices, and Good's coverage were subsequently calculated using QIIME with the default parameters (31). Principal coordinate analysis (PCoA) was performed using Bray-Curtis distance. To evaluate the effect of the dietary treatment on the microbial taxa, we used the linear discriminant analysis effect size (LEfSe) to identify different taxa using a critical value of LDA Score > 2.0 and *P*-value < 0.05 (32). To further understand the specific functions of each group of bacteria, PICRUSt2 software (33) was employed for comparing species make-up from the 16S sequencing datasets. The newly determined functional gene compositions were consequently predicted through the Kyoto Encyclopedia of Genes and Genomes (KEGG) database at level 3. Redundancy analysis (RDA) was used to analyze the relationships between differential bacteria and rumen fermentation parameters (TVFA, acetate, propionate, butyrate, valerate, and pH) on OmicStudio (LC-Bio Technology Co., Ltd., Hangzhou, China).

### Statistics and Analyses

The SPSS version 24.0 (SPSS Inc., Chicago, IL, USA) was used to analyze the data. The data of meat quality and the concentration of amino acids were analyzed by a one-way analysis of variance (ANOVA) followed by Duncan's *post-hoc* testing procedure for multiple comparisons. Alpha diversity was analyzed using Kruskal–Wallis test in the SPSS 24.0 software. Results were presented as means ± SEM. *P*-values < 0.05 were regarded as statistically significant. Correlation networks based on Spearman's rank correlation analysis between the relative abundance of key bacteria associated with carbohydrate metabolism and the meat quality indices (SF, IMF, SFA, MUFA, and PUFA) in the LT showing |*r*| > 0.60 and *P*-value < 0.05 were considered as a significant correlation.

## Results

### Effects of Different Energy Levels on the Meat Chemical Analysis and Meat Quality of the *Longissimus Thoracis* Muscle

Effects of different dietary energy levels on the LT muscle chemical composition and meat quality of yaks are shown in Tables 1, 2, respectively. The LT water and IMF content were significantly promoted (*P* < 0.05) in the HE group, compared to the LE and ME groups. However, dietary energy levels did not influence (*P* > 0.05) LT crude protein and ash content. No differences (*P* > 0.05) in the pH_1h_, pH_24h_, and meat color indices in the muscle were found among the treatments. Compared to the LE group, cooking loss and water loss of LT significantly decreased (*P* < 0.05) in the HE group, but were comparable to the ME group. The shear force was markedly reduced (*P* < 0.05) in response to the increasing dietary energy levels.

**Table 1:** Effects of dietary energy levels on the nutritional components in the *longissimus thoracis* muscle of yak.

**Items**	**Groups**			**SEM**	***P*-value**
	**LE**	**ME**	**HE**		
Water content (%)	68.94^b^	70.30^ab^	72.04^a^	0.518	0.035
Protein content (%)	23.60	23.78	24.42	0.393	0.702
IMF content (%)	0.56^c^	0.92^b^	1.34^a^	0.102	0.001
Crude ash content (%)	1.66	1.72	1.84	0.038	0.135

*LE, low energy level; ME, medium energy level; HE, high energy level; SEM, standard error of the mean; IMF, Intramuscular fat*.

*^a, b, c^Values in the same row with different superscript letters differ significantly (P < 0.05)*.

**Table 2:** Effect of dietary energy levels on the quality in the *longissimus thoracis* muscle of yak.

**Items**	**Groups**			**SEM**	***P*-value**
	**LE**	**ME**	**HE**		
pH_1h_	6.56	6.62	6.65	0.020	0.145
Ph_24h_	5.60	5.63	5.57	0.024	0.624
Cooking loss (%)	36.69^a^	32.42^ab^	28.91^b^	1.212	0.017
Driage (%)	29.49^a^	25.89^ab^	23.05^b^	1.069	0.033
Shear force (N/cm^2^)	74.50^a^	66.89^b^	55.96^c^	0.233	<0.001
CIE L*	34.22	34.88	35.29	0.250	0.222
CIE a*	18.18	18.32	18.82	0.138	0.143
CIE b*	8.16	7.88	8.24	0.087	0.226
CIE H*	24.18	23.30	23.65	0.274	0.445
CIE C*	19.94	19.94	20.54	0.132	0.094

*LE, low energy level; ME, medium energy level; HE, high energy level. SEM, standard error of the mean*.

*^a, b, c^Values in the same row with different superscript letters differ significantly (P < 0.05)*.

### Effects of Different Energy Levels on the Amino Acids Profile and Fatty Acid Composition of the *Longissimus Thoracis* Muscle

The essential amino acids (EAAs) and non-essential amino acids (NEAAs) levels in the LT muscle of yak are listed in Table 3. Methionine levels in the ME and HE groups were significantly decreased (*P* < 0.05), compared with the LE group. While, the other EAA levels were unaffected (*P* > 0.05) by the dietary energy levels. The aspartic and glycine levels in the ME and HE groups were significantly elevated (*P* < 0.05) in comparison to the LE group. Compared to the ME group, the level of glutamic acid in the LE and HE groups was lower, whereas the level of histidine in the ME group was higher than the HE group but comparable to the LE group.

**Table 3:** Effect of dietary energy levels on the amino acids content in the *longissimus thoracis* muscle of yak.

**Items**		**Groups**			**SEM**	***P*-value**
		**LE**	**ME**	**HE**		
EAA (mg/g)	Threonine	10.24	10.10	9.87	0.114	0.434
	Valine	11.77	11.56	11.08	0.155	0.185
	Methionine	0.11^a^	0.09^b^	0.09^b^	0.003	0.006
	Isoleucine	10.52	10.42	9.93	0.146	0.221
	Leucine	19.24	18.83	18.33	0.231	0.292
	Phenylalanine	9.96	9.50	9.59	0.145	0.426
	Lysine	21.52	21.09	20.47	0.258	0.263
NEAA (mg/g)	Aspartic acid	24.94^a^	22.90^b^	23.05^b^	0.327	0.006
	Glutamic acid	36.58^b^	38.82^a^	35.03^b^	0.534	0.004
	Cystine	8.01	7.15	7.73	0.161	0.074
	Serine	9.60	9.28	9.19	0.104	0.243
	Glycine	10.19^a^	9.14^b^	8.90^b^	0.221	0.023
	Histidine	8.59^ab^	9.12^a^	8.34^b^	0.130	0.030
	Arginine	13.96	13.92	13.28	0.162	0.154
	Alanine	13.27	12.86	12.68	0.140	0.220
	Proline	8.21	7.81	7.72	0.110	0.159
	Tyrosine	8.11	7.78	7.58	0.105	0.114

*LE, low energy level; ME, medium energy level; HE, high energy level; EAA, essential amino acids; NEAA, non-essential amino acids; SEM, standard error of the mean*.

*^a, b, c^Values in the same row with different superscript letters differ significantly (P < 0.05)*.

The fatty acid composition of the LT muscle is listed in Supplementary Table S2, which was previously published (18). Briefly, no differences were observed (*P* > 0.05) in the total monounsaturated fatty acid (MUFA) and polyunsaturated fatty acids (PUFA) between the LE and ME groups, while the proportions of saturated fatty acids (SFA) in the ME and HE groups were significantly higher (*P* < 0.05) than the LE group. The concentrations of SFA in the HE group was markedly elevated compared to the ME group. In this study, MUFA and PUFA levels in the HE group were markedly elevated compared to the other groups (*P* < 0.05).

### Diversity Taxonomy and Function Prediction of Rumen Bacteria

**Figure 1:**
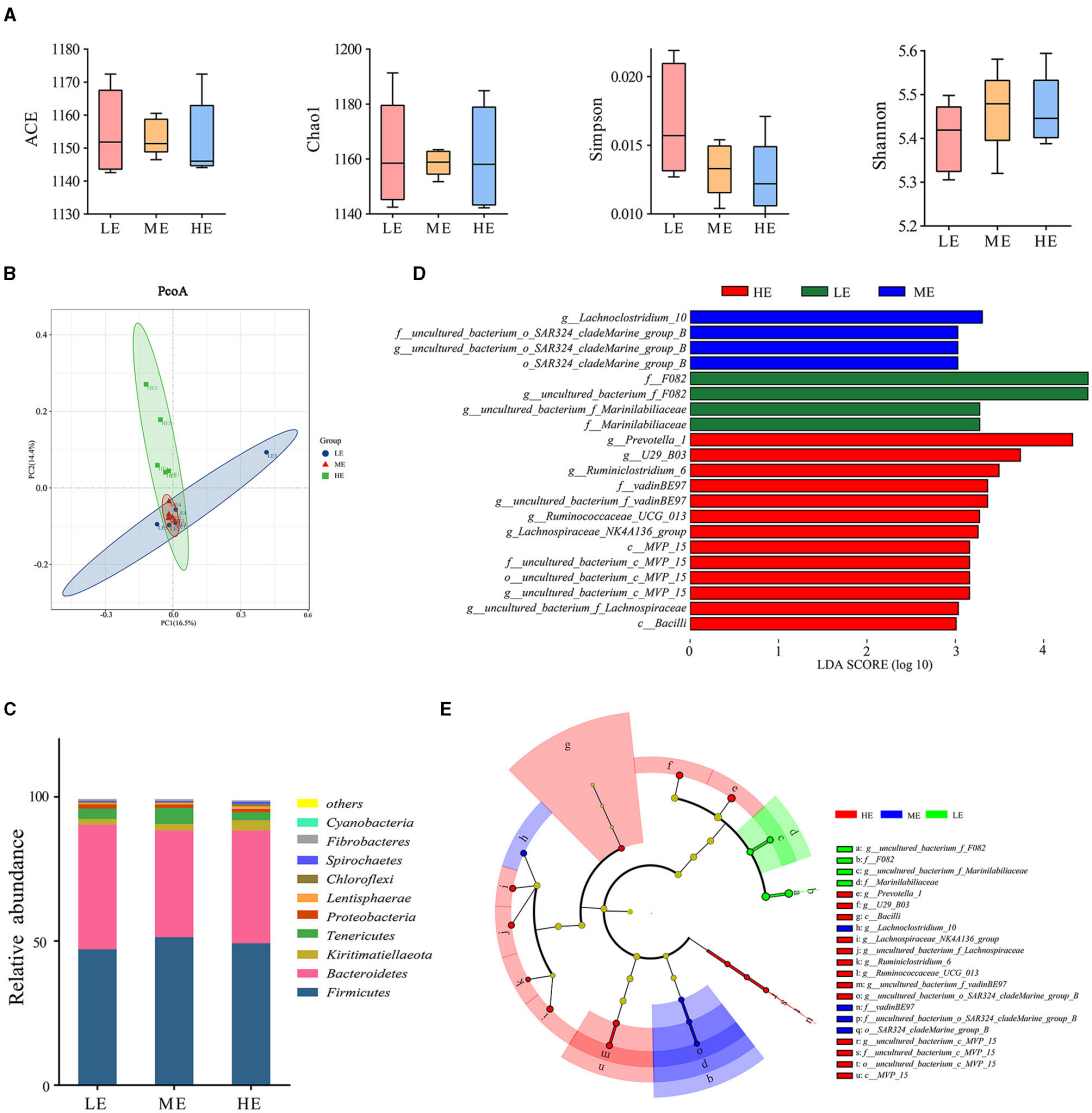
Effect of dietary energy level on the diversity and structure of ruminal bacteria. **(A)** Differences in yak ruminal bacterial diversity and richness between the LE, ME, and HE groups. Box plot showing the alpha diversity of the rumen bacterial communities in yaks given different dietary energy levels. **(B)** Principal-coordinate analysis (PCoA) of bacterial communities based on OTUs. **(C)** Effects of dietary energy levels on the yak rumen bacterial composition at the phylum level. Each bar and color represent the average relative abundance of each phylum, and the 10 most abundant taxa are shown. **(D)** Linear Discriminant Analysis Effect Size (LEfSe). Histogram of the LDA scores for differentially abundant genera among the HE, ME, and LE groups (LDA score ≥ 3). **(E)** A cladogram showing the differences in the relative abundance of the taxa at five levels between the HE, ME, and LE groups.

In this study, the unannotated reads were only 0.30% at the genus level after re-annotating the OTU based on the SILVA database compared to 33.83% unannotated reads of the previous study annotated by the GreenGene Database (Supplementary Table S3). Microbial abundance and heterogeneity were assessed through the alpha diversity indices. Alpha diversity measures revealed that different dietary energy levels have a little effect on (*P* > 0.05) the number of OTUs, the ACE and Chao1 estimator, and the Shannon index (Figure 1A). The PCoA based on the Bray-Curtis distance showed that the HE group presented a degree of diversity discrepancy with the other two groups (Figure 1B), indicating that the rumen bacterial community changed significantly with the increase of dietary energy. In addition, 19 phyla were discovered in the rumen for all groups (Figure 1C). *Firmicutes, Bacteroidetes, Kiritimatiellaeota*, and *Tenericutes* were the dominant phyla across all groups. *Firmicutes* and *Bacteroidetes* were the most dominant phyla, accounting for 88% of the total population. Concomitantly, 212 classifiable genera were detected in all samples. The results of LEfSe analysis at the genus level are shown in Figures 1D,E. In detail, the rumen bacteria in the ME group were mainly composed of *Lachnoclostridium_10* and *uncultured_bacterium_o_SAR324_cladeMarine_group_B* at the genus level, while *uncultured_bacterium_f_F082* and *uncultured_bacterium_f_Marinilabiliaceae* were significantly (*P* < 0.05) more abundant in the LE group. In addition, eight bacterial taxa including *uncultured_bacterium_f_Lachnospiraceae, uncultured_bacterium_c_MVP_15, Lachnospiraceae_NK4A136_group, U29_B03, uncultured_bacterium_f_vadinBE97, Ruminiclostridium_6, Prevotella_1*, and *Ruminococcaceae_UCG_013* were overrepresented (*P* < 0.05) in the HE group at the genus level.

**Figure 2:**
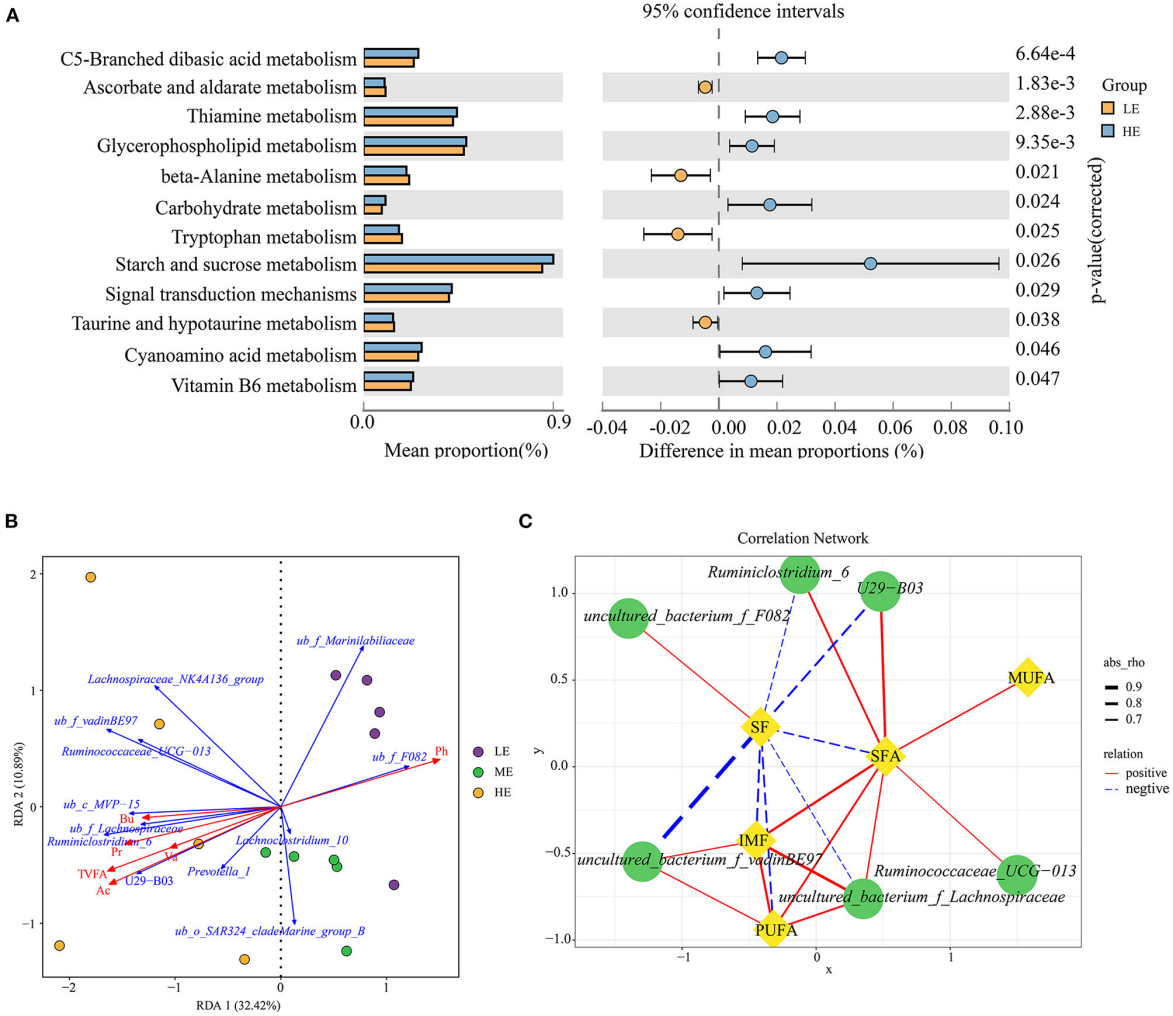
Predicted function analysis of ruminal bacteria and correlation analysis between the phenotypic values and ruminal main taxa. **(A)** Pathways were predicted by PICRUSt2. The significant microbial metabolic pathways between the HE and LE groups (*P*-value < 0.05) are shown. **(B)** Redundancy analysis (RDA) of the sequencing data of the 16S rRNA gene showing relationships between the differential bacteria and environmental factors (TVFA, acetate, propionate, butyrate, valerate, and pH). TVFA, Total VFA; Ac, Acetate; Pr, Propionate; Bu, Butyrate; Va, valerate; ub, uncultured_bacterium. **(C)** Spearman's correlation network showing relationships between the relative abundance of differential bacteria at the genus level and the meat quality indices. Only the strong correlations (|*r*| > 0.60 and *P*-value < 0.05) were showed in the correlation networks.

PICRUSt2 was used to investigate the possible microbial metabolic pathways. As shown in Figure 2A, the mean proportion of starch and sucrose metabolism was the highest in the rumen microbial metabolism pathway, and was significantly higher in the HE group than in the LE group (*P* = 0.026). Meanwhile, carbohydrate metabolism was also higher (*P* = 0.024) in the HE group than in the LE group based on the level 3. The proportion of metabolic pathways at level 3 between the LE and ME groups had no difference (*P* > 0.05), while the abundance of significantly different metabolic pathways between the ME and HE groups were lower than 0.25%, therefore, the results of these two comparisons did not present.

### The Relationship Between the SF, IMF of *Longissimus Thoracis*, and the Relative Abundance of Differential Bacteria, Phenotype of Fatty Acid Profile

To illustrate the relationship between the significant taxa and ruminal fermentation parameters that were published previously (the data are presented in Supplementary Table S4) (24), and an RDA ranking map was generated (Figure 2B). In detail, the bacterial genera *uncultured_bacterium_f_Marinilabiliaceae, Lachnoclostridium_10*, and *uncultured_bacterium_f_F082* exhibited a positive relationship with the pH value, and showed a negative relationship with the total VFA, acetate, propionate, butyrate, and valerate. The relative abundance of *Prevotella_1, U29_B03, Ruminiclostridium_6, uncultured_bacterium_f_Lachnospiraceae, uncultured_bacterium_c_MVP_15, Ruminococcaceae_UCG_013, uncultured_bacterium_f_vadinBE97*, and *Lachnospiraceae_ NK4A136_group* was positively associated with the total VFA, acetate, propionate, butyrate, and valerate.

The correlation results revealed 20 significant Spearman's correlations between differential bacteria and meat quality traits (SF and IMF) as well as fatty acid profile (SFA, MUFA, and PUFA) (*R* > 0.60, *P* < 0.05, Figure 2C). In detail, the SF had a negative relationship with *Ruminiclostridium_6, U29_B03, uncultured_bacterium_f_vadinBE97, uncultured_ bacterium_f_Lachnospiraceae*, SFA, and PUFA, and a positive relationship with *uncultured_bacterium_f_F082*. The IMF exhibited a significantly positive correlation with *uncultured_bacterium_f_Lachnospiraceae, uncultured_ bacterium_f_vadinBE*97, and PUFA. In addition, the SFA was positively correlated with *Ruminiclostridium_6, U29_B03, Ruminococcaceae_UCG_013, uncultured_bacterium_f_Lachnospiraceae*, IMF, MUFA, and PUFA. The PUFA had a positive relationship with *uncultured_bacterium_f_Lachnospiraceae* and *uncultured_ bacterium_f_vadinBE*97.

## Discussion

In the current study, the effects of dietary energy level on the phenotypic parameters related to the meat quality in the LT muscle of yak were focused. Meanwhile, the relationships between meat quality and ruminal bacteria were illustrated, and the contribution of bacteria on fatty acid synthesis in the LT muscle through generating substrates (mainly volatile fatty acids) was assessed. These findings gained a comprehensive understanding of the regulatory mechanisms of the improvement of the meat quality induced by different dietary energy levels.

It is necessary to have a detailed understanding of the physical and chemical properties (e.g., pH, color), as well as the storage quality of meat to determine the quality of meat after slaughtering (34). The pH value of meat is an essential factor that influences the color, tenderness, cooking loss, shelf-life, and other physicochemical properties (35). After slaughtering, the breakdown of glycogen in the muscles results in the accumulation of a large amount of lactic acid which led to a pH reduction of the meat to an ultimate pH value at 24 h (36). Previous studies demonstrated that the optimal range of pH_24h_ for beef cattle is between 5.4 and 5.6 (37); once the pH_24h_ value is higher than 6.0, the meat tends to be dark, firm, and dry (DFD) (38). In this experiment, dietary energy levels showed no impact on the pH_24h_ of the LT muscle and the pH values of yak meat in each group were within or near to the aforementioned optimal range, suggesting that high-quality yak meat in each group was earned. Cooking loss and water holding capacity are important factors in evaluating the meat quality, which affect the juiciness of cooked meat (39). In the present study, the significant decreasing trend of cooking loss and water loss were observed with increasing dietary energy levels, which is consistent with the study of Kang et al. (6). Shear force is an important indicator of meat tenderness. The tenderness of the meat is a major characteristic that is highly related to the overall acceptability of consumers of yak meat. It is highly variable and can be affected by many factors, including muscle fiber temperament, connective tissue composition, and protease configuration modulations within the muscle mass (40). In this study, shear forces were decreased with increasing dietary energy levels. It falls outside the optimum range proposed by Miller et al. (41). The discrepancy in the results might be due to the difference in the diet, age, and breed (42). Zeng et al. (43) have demonstrated that a drastic reduction in shear force due to elevated dietary energy levels could be attributed to an enhanced IMF content. IMF affects the modification of muscle fiber condition, the composition, and content of connective tissue in the muscle, and the configuration of protease in muscle, which can affect muscle tenderness (44). Our results showed that high-energy diets resulted in an elevated IMF content, which can reduce collagen cross-linking and contributes to the tenderness of the meat (45). Liu (46) and Hwang et al. (47) reported that the IMF content has a positive correlation with meat tenderness and juiciness. Furthermore, in this trial, the water content and IMF content in the LT muscle elevated with the increase in the dietary energy level. Therefore, we speculated that the difference in yak meat tenderness receiving different dietary energy levels could be explained through the IMF variations and fatty acid composition. It has been reported that the rumen bacteria could indirectly affect the metabolite deposits within the muscle-mass due to their interplays with the host organism (48).

Amino acids are the basic components of animal protein, and the changes in the amino acid composition directly affect the nutritional value of the meat (49). Since EAA could not be synthesized *in vivo*, the difference in the methionine content in the LT muscle for each group might be caused by differences in the dietary methionine content. The rumen microbiome is essential for meat generation, with rumen microbial protein being a major precursor for meat protein (50). The contents of aspartic acid, glycine, glutamic acid, and histidine might be affected by the microbial synthesis in the rumen. *Streptococcus bovis, Selenomonas ruminantium*, and *Prevotella bryantii* of rumen microorganisms are reported to be involved in the *de novo* synthesis of amino acids (51). In this study, dietary energy levels significantly affected the relative abundance of *Prevotella*. Generally, the levels of glutamate/glutamine in beef were the highest, accounting for 16.5% of the total amino acids, followed by aspartic (52), which is consistent with the results of the present study. The increased glutamic acid production in the rumen of ruminants can increase glutamic acid synthesis, and glutamine can be converted into glucose *in vivo* (53). Therefore, the increase of glutamic acid content in the LT muscle of yak fed with a medium dietary energy level might be due to the contribution of rumen microorganisms.

In the current study, neither the alpha diversity nor the relative abundances of the main phyla showed significant variations among the different dietary treatments. The relative abundances of the *Firmicutes* and *Bacteroidetes* were observed to be important phyla in the three groups. At the genus level, the majority of the genera present in all groups were not affected by the different diets, which is consistent with the results reported by Bi et al. (54). Interestingly, most of the differential bacteria at the genus level belonged to the carbohydrate-degrading bacteria. Rumen microbes degrade carbohydrates into volatile fatty acids (VFAs) to provide 70–80% of the metabolizable energy (55), which are the main substrates for the synthesis of milk fat and body fat (56). Genus *Prevotella_1* is a dominant beneficial bacterial species, which plays a vital role in the degradation of starch, xylan, protein, peptide, hemicellulose, and pectin (57). In the present study, the abundance of *Prevotella_1* increased with the level of starch in the diet, which is consistent with a previous study in Holstein-Friesians bulls (58). In the current study, *Prevotella_1* had the highest LDA score in the HE group, which is mainly involved in the fermentation of starch and production of propionic acid. *U29_B03* is a member of the phylum Bacteroidetes belonging to the family *Rikenellaceae*, and was found to be involved in the degradation of structural carbohydrates (59). In addition, a previous study has reported that the relative abundance of *Rikenellaceae* was elevated in humans and mice when fed a diet with a high resistant starch level (60). Similarly, the relative abundance of *U29_B03* was found to be significantly higher in the HE group than in the ME and LE groups, which indicates that the genus *U29_B03* might be involved in carbohydrate degradation, especially starch metabolism. The genus *uncultured_bacterium_f_vadinBE97*, which belongs to efficient sugar-fermenting families (*vadinBE97*) (61), was significantly higher in the HE group than in the other groups. The bacterial families *Lachnospiraceae* and *Ruminococcaceae* are known to produce butyrate by degrading complex polysaccharides, including starch (62), which supported our results of a dramatic increase in the abundance of four genera (*uncultured_bacterium_f_Lachnospiraceae, Lachnospiraceae_NK4A136_group, Lachnospiraceae_10, Ruminiclostridium_6*, and *Ruminococcaceae_UCG_013*) which belonged to the families *Lachnospiraceae* and *Ruminococcaceae*. The *uncultured_bacterium_c_MVP_15* is rare in the rumen and its function needs to be studied further; however, its bacterial phylum (Spirochaetes) has shown that it is primarily responsible for the degradation of starch in a starch-fed reactor (63). In the current study, we observed a decreased abundance of *uncultured_bacterium_f_F082* with increasing dietary energy levels, which was consistent with the results of Zened et al. (11) that uncultured or unclassified bacteria in the rumen were negatively affected by starch addition.

Microbial potential function analysis with PICRUSt2 indicated that the most prominent functional categories were starch and sucrose metabolism and carbohydrate metabolism at the KEGG level 3 metabolic category. As we expected, the proportion of starch metabolism was higher in the HE group than in the LE group when the dietary energy levels increased (corn as the main energy source and the starch content increased with the dietary energy level). Actually, rumen microbial degradation of dietary starch elevated the propionic acid concentration with the increase of starch content. Propionic acid is mainly used in the gluconeogenesis of the liver to synthesize glucose. Propionic acid and glucose are substrates of long-chain fatty acid esterification and fat formation in IMF (64). In the present study, the IMF was found to be significantly positively corrected with SFA and PUFA, probably the high degree of biohydrogenation of unsaturated fatty acids in the rumen and the contribution of SFA to intramuscular fat deposition was greater than that of unsaturated fatty acids (48). Genus *uncultured_bacterium_f _vadinBE97* and *uncultured_bacterium_f_Lachnospiraceae* had a positive influence on the content of IMF, indicating these bacteria underwent a rapid proliferation with the increase in substrates to degrade dietary carbohydrate and provide VFAs to IMF deposition. *Ruminiclostridium_6* and *U29-B03* had a positive influence on the SFA and VFAs had a negative influence on the SF, suggesting that the above-mentioned bacteria may patriciate might participate in carbohydrate metabolism to produce VFAs, thereby facilitating IMF deposition to promote tenderness in the LT muscle. *Ruminococcaceae_UCG-013* genus is famous for its ability to degrade cellulose and hemicellulose in the rumen and produce butyric acid (65). However, in this study, *Ruminococcaceae_UCG-013* had a strong positive correlation with butyric acid and SFA, suggesting that *Ruminococcaceae_UCG-013* was tolerant to a high starch diet. In summary, the aforementioned bacteria mainly degraded dietary starch into VFAs that provided substrates for fatty acid synthesis and, finally, accelerated the fat deposition and enhanced the meat quality in the LT muscle.

It must be taken into account that the exploration of rumen microbial degradation products might be associated with the observed phenotypic differences in the LT muscle. However, the underlying mechanism is still not well-known and more research is needed. Another limitation of this study is that the sample size was too small because yaks are important and expensive for herders, so there is no way to expand the sample size.

## Conclusions

Feeding different energy level diets improved fat deposition, water holding capacity, and tenderness and changed the content of functional amino acids in the LT muscle of yak. Moreover, the carbohydrate metabolism-related bacteria, especially amylolytic bacteria, were positively correlated with the content of IMF but had a negative impact on SF. The results indicated that high dietary energy levels could improve the meat quality in the LT muscle of yak through increasing the abundance of amylolytic bacteria and their fermentation products to provide substrates for fatty acid synthesis.

## Data Availability Statement

Supplementary Material and Supporting Data for this article are depositied in the database and can be found at can be found online a https://www.jianguoyun.com/p/DbXaVqwQmrzKCRietoUE. The raw data of 16s rDNA sequence for rumen bacteria of yaks in LE, ME and HE groups were obtained from our previously study (24), and the raw sequence data have been deposited in the European Nucleotide Archive (ENA) at EMBL-EBI under accession number PRJEB34298.

## Ethics Statement

The animal study was reviewed and approved by the procedures of animal experiments complied with Gansu Province Animal Care Committee (Lanzhou, China), and experimental protocols in this study were reviewed and approved by the Animal Care and Use Committee of Lanzhou Institute of Husbandry and Pharmaceutical Sciences, China [SCXK (GAN) 2014-0002]. Written informed consent was obtained from the owners for the participation of their animals in this study.

## Author Contributions

XD and CY designed the manuscript. MD wrote the manuscript. XD, PY, YY, JZ, AA, and ZL provided the writing guidance and revised the manuscript. All authors contributed to the article and approved the submitted version.

## Funding

This work was supported by a grant from the International Cooperation and Exchange Program of the National Natural Science Foundation of China (31461143020) and the Innovation Program of Chinese Academy of Agricultural Sciences (00&0656).

## Conflict of Interest

The authors declare that the research was conducted in the absence of any commercial or financial relationships that could be construed as a potential conflict of interest.

## Publisher's Note

All claims expressed in this article are solely those of the authors and do not necessarily represent those of their affiliated organizations, or those of the publisher, the editors and the reviewers. Any product that may be evaluated in this article, or claim that may be made by its manufacturer, is not guaranteed or endorsed by the publisher.
