# Supplementary material for: Dietary Energy Levels Affect Carbohydrate Metabolism-Related Bacteria and Improve Meat Quality in the Longissimus Thoracis Muscle of Yak (Bos grunniens)
Source: Front Vet Sci. 2021 Sep 22;8:718036. doi: 10.3389/fvets.2021.718036 (PMC8492897; doi:10.3389/fvets.2021.718036)
Supplement: Supplementary file 1 [file Table_1.DOCX]

**TABLE S1**. Ingredients and nutrient composition of three concentrates used during the experiment.

| Items | Groups^1^ | | |
| --- | --- | --- | --- |
|  | LE | ME | HE |
| Ingredient, (%) of DM | | | |
| Corn | 29.00 | 44.80 | 56.00 |
| Corn germ | 30.00 | 20.00 | 12.00 |
| Wheat bran | 4.00 | 4.00 | ─ |
| DDGS^2^ | 15.00 | 7.00 | 6.30 |
| Prickly ash seed | 10.00 | 2.00 | 4.00 |
| Cottonseed meal | 6.00 | 12.00 | 16.00 |
| Soybean meal | ─ | 5.00 | ─ |
| Salt | 0.80 | 0.80 | 0.80 |
| White stone powder | 2.00 | 2.00 | 2.00 |
| Dicalcium phosphate | 0.60 | 0.60 | 0.60 |
| Urea | 0.80 | ─ | 0.50 |
| Sodium bicarbonate | 1.00 | 1.00 | 1.00 |
| Premix^3^ | 0.80 | 0.80 | 0.80 |
| Nutrient composition, g/kg of DM (%) | | | |
| Crude protein | 16.53 | 16.74 | 17.21 |
| Crude fat | 3.73 | 4.18 | 5.57 |
| NEg^4^ (MJ/kg) | 5.5 | 6.2 | 6.9 |
| Neutral detergent fiber | 15.93 | 13.15 | 12.32 |
| Acid detergent fiber | 4.54 | 4.14 | 3.72 |
| Calcium | 0.64 | 0.84 | 0.75 |
| Phosphorus | 0.31 | 0.34 | 0.36 |

^1^ LE, low energy level; ME, medium energy level; HE, high energy level.

^2^ DDGS, distillers dried grains with solubles.

^3^ Premix was provided per kilogram of total diet DM, and the composition was as follows: 22,520 IU of vitamin A, 1920 IU of vitamin D3, 18 IU of vitamin E, 0.36 IU of vitamin K3, 5.28 mg of vitamin B2, 0.008 mg of vitamin B12, 21.2 mg of D-calcium pantothenate, 9 mg of Cu, 132.8 mg of Zn, 240 mg of Fe, 8 mg of Mn, and 0.28 mg of Co.

^4^NEg, net energy for gain, is calculated according to Feeding Standard of Beef Cattle (NY/T 815-2004); others were measured values.

| Items | Groups^1^ | | | SEM | P-value |
| --- | --- | --- | --- | --- | --- |
|  | LE | ME | HE |  |  |
| C14:0 | 15.35^c^ | 21.55^b^ | 25.11^a^ | 1.138 | <0.001 |
| C15:0 | 2.08^b^ | 3.01^ab^ | 4.03^a^ | 0.308 | 0.020 |
| C16:0 | 1951.06^c^ | 2748.31^b^ | 3367.25^a^ | 174.237 | <0.001 |
| C17:0 | 6.31^b^ | 7.81^b^ | 10.26^a^ | 0.595 | 0.010 |
| C18:0 | 1044.74^c^ | 1242.88^b^ | 1588.32^a^ | 63.775 | <0.001 |
| SFA | 3019.55^c^ | 4023.57^b^ | 4994.97^a^ | 232.906 | <0.001 |
| C14:1 | 3.33^c^ | 4.14^b^ | 5.73^a^ | 0.291 | <0.001 |
| C16:1 | 69.80^c^ | 81.91^b^ | 118.14^a^ | 5.60 | <0.001 |
| C17:1 | 5.20^b^ | 6.02^ab^ | 8.44^a^ | 0.566 | 0.038 |
| C18:1n9C | 1952.68^b^ | 2441.40^ab^ | 3059.18^a^ | 186.659 | 0.038 |
| C18:1n9T | 37.37^b^ | 44.45^b^ | 75.05^a^ | 5.866 | 0.008 |
| C20:1 | 1.22^b^ | 1.82^a^ | 2.11^a^ | 0.136 | 0.012 |
| MUFA | 2069.60^b^ | 2579.74^ab^ | 3268.65^a^ | 195.509 | 0.027 |
| C18:2n6C | 64.49^b^ | 66.39^b^ | 80.41^a^ | 2.846 | 0.029 |
| C20:3n3 | 0.71^b^ | 1.14^a^ | 1.31^a^ | 0.085 | 0.002 |
| C20:4n6 | 8.61^c^ | 11.81^b^ | 18.81^a^ | 1.185 | <0.001 |
| C20:5n3 | 1.84^b^ | 2.22^ab^ | 3.08^a^ | 0.212 | 0.036 |
| C22:6n3 | 1.26^b^ | 1.54^ab^ | 2.17^a^ | 0.157 | 0.038 |
| PUFA | 76.91^b^ | 83.09^b^ | 105.78^a^ | 4.134 | 0.002 |

**TABLE S2**. Fatty acid composition in longissimus dorsi of yaks fed diets supplying different energy levels.

^1^LE, low energy level; ME, medium energy level; HE, high energy level. SEM, standard error of the mean. a, b, c Means in a row with different small letter superscripts differ significantly (P < 0.05). SFA, saturated fatty acid; MUFA, monounsaturated fatty acid; PUFA, polyunsaturated fatty acid.
