# Supplementary material for: Dietary Energy Levels Affect Carbohydrate Metabolism-Related Bacteria and Improve Meat Quality in the Longissimus Thoracis Muscle of Yak (Bos grunniens)
Source: Front Vet Sci. 2021 Sep 22;8:718036. doi: 10.3389/fvets.2021.718036 (PMC8492897; doi:10.3389/fvets.2021.718036)
Supplement: Supplementary file 3 [file Table_3.DOCX]

**Supplementary table3.** Effects of different dietary energy levels on rumen VFA concentrations of yak.

| Items | Groups^1^ | | | SEM | P-value |
| --- | --- | --- | --- | --- | --- |
|  | LE | ME | HE |  |  |
| pH | 6.90^a^ | 6.66^b^ | 6.47^c^ | 0.05 | <0.001 |
| Total VFA (mM) | 51.4^c^ | 60.9^b^ | 72.1^a^ | 2.41 | <0.001 |
| Acetate (mM) | 37.4^c^ | 44.9^b^ | 51.9^a^ | 1.71 | <0.001 |
| Propionate (mM) | 8.7^c^ | 9.7^b^ | 11.4^a^ | 0.36 | 0.001 |
| Butyrate (mM) | 4.02^c^ | 4.98^b^ | 7.11^a^ | 0.45 | <0.01 |
| Isobutyrate (mM) | 0.32 | 0.33 | 0.37 | 0.01 | 0.08 |
| Valerate (mM) | 0.26^c^ | 0.31^b^ | 0.44^a^ | 0.02 | <0.01 |
| Isovalerate (mM) | 0.66 | 0.66 | 0.77 | 0.02 | 0.09 |
