# Supplementary material for: Dietary Energy Levels Affect Carbohydrate Metabolism-Related Bacteria and Improve Meat Quality in the Longissimus Thoracis Muscle of Yak (Bos grunniens)
Source: Front Vet Sci. 2021 Sep 22;8:718036. doi: 10.3389/fvets.2021.718036 (PMC8492897; doi:10.3389/fvets.2021.718036)
Supplement: Supplementary file 4 [file Table_4.DOCX]

Raw Data Supplementary Information file

The raw data of 16s rDNA sequence for rumen bacteria of yaks in LE, ME and HE groups have been deposited in the European Nucleotide Archive (ENA) at EMBL-EBI under accession number PRJEB34298.

All the original source data (raw data, original data, individual data points) which are presented in tables and figures with Excel version are uploaded to the specified website and the download links as follow: https://www.jianguoyun.com/p/DbXaVqwQmrzKCRietoUE
